# Detecting Functional Divergence after Gene Duplication through Evolutionary Changes in Posttranslational Regulatory Sequences

**DOI:** 10.1371/journal.pcbi.1003977

**Published:** 2014-12-04

**Authors:** Alex N. Nguyen Ba, Bob Strome, Jun Jie Hua, Jonathan Desmond, Isabelle Gagnon-Arsenault, Eric L. Weiss, Christian R. Landry, Alan M. Moses

**Affiliations:** 1 Department of Cell & Systems Biology, University of Toronto, Toronto, Canada; 2 Centre for the Analysis of Genome Evolution and Function, University of Toronto, Toronto, Canada; 3 Département de Biologie, IBIS and PROTEO, Pavillon Charles-Eugene-Marchand, Laval University, Québec City, Canada; 4 Department of Molecular Biosciences, Northwestern University, Evanston, Illinois, United States of America; Ghent University, Belgium

## Abstract

Gene duplication is an important evolutionary mechanism that can result in functional divergence in paralogs due to neo-functionalization or sub-functionalization. Consistent with functional divergence after gene duplication, recent studies have shown accelerated evolution in retained paralogs. However, little is known in general about the impact of this accelerated evolution on the molecular functions of retained paralogs. For example, do new functions typically involve changes in enzymatic activities, or changes in protein regulation? Here we study the evolution of posttranslational regulation by examining the evolution of important regulatory sequences (short linear motifs) in retained duplicates created by the whole-genome duplication in budding yeast. To do so, we identified short linear motifs whose evolutionary constraint has relaxed after gene duplication with a likelihood-ratio test that can account for heterogeneity in the evolutionary process by using a non-central chi-squared null distribution. We find that short linear motifs are more likely to show changes in evolutionary constraints in retained duplicates compared to single-copy genes. We examine changes in constraints on known regulatory sequences and show that for the Rck1/Rck2, Fkh1/Fkh2, Ace2/Swi5 paralogs, they are associated with previously characterized differences in posttranslational regulation. Finally, we experimentally confirm our prediction that for the Ace2/Swi5 paralogs, Cbk1 regulated localization was lost along the lineage leading to *SWI5* after gene duplication. Our analysis suggests that changes in posttranslational regulation mediated by short regulatory motifs systematically contribute to functional divergence after gene duplication.

## Author Summary

How a protein is controlled is intimately linked to its function. Therefore, evolution can drive the functional divergence of proteins by tweaking their regulation, even if enzymatic capacities are preserved. Changes in posttranslational regulation (protein phosphorylation, degradation, subcellular localization, etc.) could therefore represent key mechanisms in functional divergence and lead to different phenotypic outcomes. Since disordered protein regions contain sites of protein modification and interaction (known as short linear motifs) and evolve rapidly relative to domains encoding enzymatic functions, these regions are good candidates to harbour sequence changes that underlie changes in function. In this study, we develop a statistical framework to identify changes in rate of evolution specific to protein regulatory sequences and identify hundreds of short linear motifs in disordered regions that are likely to have diverged after the whole-genome duplication in budding yeast. We show that these divergent motifs are much more frequent in paralogs than in single-copy proteins, and that they are more frequent in duplicate pairs that have functionally diverged. Our analysis suggests that changes in short linear motifs in disordered protein regions could be important molecular mechanisms of functional divergence after gene duplication.

## Introduction

Gene duplication is thought to be one of the major sources of evolutionary innovation (reviewed in [1]). Several molecular mechanisms of functional change have been proposed: 1) changes at the transcriptional level can alter the expression of the paralogous copy [2]–[5], 2) changes at the enzymatic level can alter the activity or specificity of the protein [1], [6], 3) changes at the posttranslational level can modify the regulation or localization of the protein [7]–[9], and 4) changes within the splicing sites can change the isoforms produced at each loci [10], [11]. Studies on genome-wide mRNA expression patterns have established that transcriptional changes are one of the major contributors of functional differences within duplicated genes [12]–[14]. However, whether functional divergence occurs predominantly by changes in gene regulation or by changes within the amino acid coding sequence of the proteins are still unclear [15].

Coding sequences of paralogous genes show increased evolutionary rates after duplication [16], [17], consistent with the hypothesis that changes within the amino acid coding sequences are also important contributors to functional divergence. However, because some functional features in proteins comprise a small number of amino acids, statistical studies comparing evolutionary rates of whole proteins do not provide mechanistic explanations for changes in function [18]. For example, many proteins contain short linear motifs (SLiMs) such as phosphorylation sites, localization signals and interaction motifs, and these motifs are only 2-15 amino acids long [19]. For instance, the cell-cycle regulator Sic1 is a disordered protein with several phosphorylation and protein binding sites that comprise less than 20% of the protein [20]. Computational identification of short linear motifs is an important challenge, often relying on experimental data [21], [22]. However, recently we [23] and others [24] have shown that they can be systematically identified in fast evolving disordered regions because they tend to be preferentially conserved. Nevertheless, most short linear motifs in disordered regions probably remain uncharacterized [23]. Therefore, analyses on whole proteins may underestimate the level of functional divergence after gene duplication because changes in constraints in short linear motifs may lead to regulatory changes and therefore functional divergence [8]. Recently, several studies have investigated specific types of posttranslational regulatory changes [8], [25]–[27] (reviewed in [28]), such as differences in patterns of phosphorylation between paralogs [9] or differences in localization in paralogous proteins [7], and have shown that regulatory changes can also contribute to functional divergence. However, these regulatory changes can also be attributed in part to *trans*-regulatory changes (changes in proteins that control posttranslational regulation). Identification of changes in the protein regulatory sequences would allow us to determine *cis*-regulatory divergence (changes within duplicated proteins), and provide amino acid level mechanistic explanations for protein regulatory changes after duplication [29].

Formally, functional divergence in amino acid sequences after gene duplication has been divided into two types of evolution [30]. The first (type I) describes so-called “changes in constraint” where the rate of evolution in a site or region changes after duplication, and remains different in one of the paralogous clades. The second (type II) describes a burst of rapid evolution immediately after gene duplication, and then a restoration of similar levels of constraint in the two paralogous lineages. Several statistical methodologies have been developed to identify sites or regions in proteins that fall into these classes [31], [32]. These approaches have largely focused on identifying sites in globular regions of proteins for which large numbers of homologues can be accurately aligned [33]. These approaches often use likelihood-ratio tests based on advanced probabilistic models of phylogeny and amino acid substitution to compare the rates of evolution in individual sites [34] or groups of sites [31], [32] to the rest of the protein. For example, previous applications of these methods have identified possible positions in the globular domain of carbonic anhydrase III that are responsible for posttranslational addition of glutathione [35]. In principle, these methods could be applied to identify changes in short linear motifs within disordered regions that contribute to posttranslational regulatory change. However, because real protein evolution can be more complicated than even the most sophisticated models [36] and real protein alignments include non-biological sources of heterogeneity (such as alignment errors and missing data), the likelihood-ratio test can falsely identify type I functional divergence [32]. One strategy to tackle these issues is to estimate the rejection rate of the likelihood-ratio test using empirical data, for example using permutation tests [37]. However, the distribution of the likelihood-ratio test statistic must be obtained through permutations performed for every protein and therefore may be too laborious for genome-wide studies.

We set out to study the change in selective constraints in short linear motifs within disordered regions after the whole-genome duplication (WGD) in budding yeast by asking whether the rates of evolution of these segments significantly differed after the whole-genome duplication event. We first developed a statistical method to correct the p-value distributions of likelihood-ratio tests and show how this approach can be applied to predicted short linear motifs. We then show that the turnover of predicted motifs within retained paralogs is faster than in genes whose paralogs were lost after duplication (which we refer to as single-copy genes or proteins) and that, for these putative short linear motifs, correlated loss of selective constraints appear to be common, consistent with changes in function specific to one of the two paralogs.

Finally, we identify examples of experimentally verified motifs present in one paralog that are unlikely to be present in the other copy, and verify our prediction of changes in subcellular localization for one of these examples (Ace2 and Swi5). Our results show that a view of molecular evolution with amino acid resolving power can allow us to propose specific hypotheses about the functional divergences between paralogs.

## Results

### Detection of type I functional divergence in short linear motifs using a non-central chi-squared null distribution for likelihood-ratio tests

**Figure 1:**
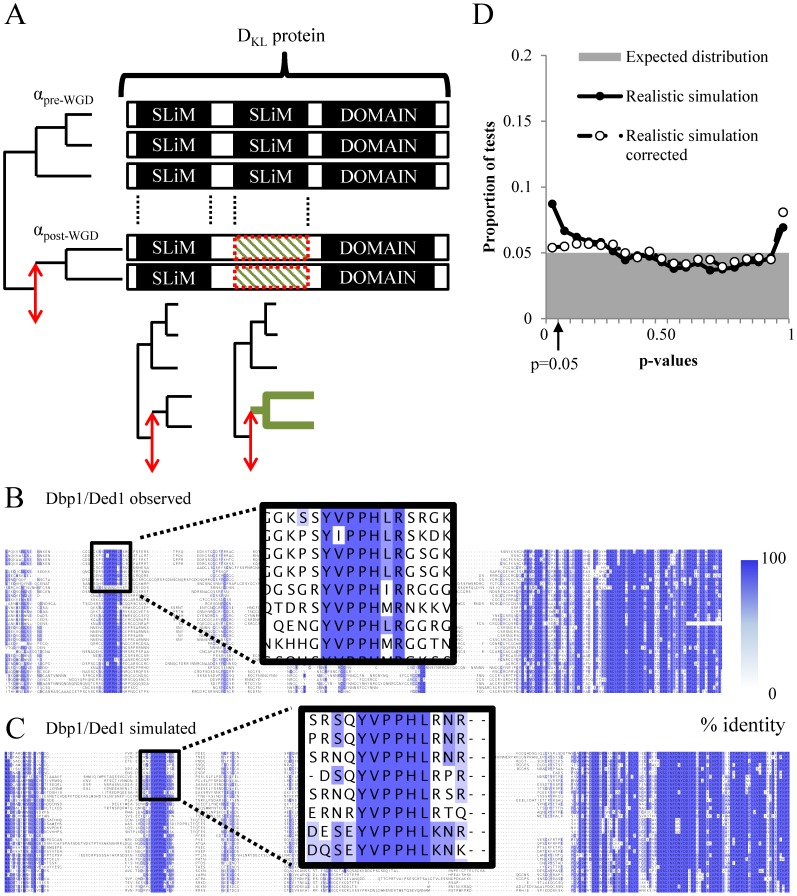
Likelihood-ratio test on short linear motifs after gene duplication on simulated data. A) Schematic of the motif-specific likelihoodratio test applied to all motifs. Rates of evolution are computed for each motif before (α_pre-WGD_) and after (α_WGD_) gene duplication and compared with the rates that were observed for the whole protein (see Methods). Red double arrow illustrates the duplication event. Bolded clades are clades with significant changes in constraints. Striped patterned boxes indicate short linear motifs with significantly different rate of evolution. D_KL_ indicates the expected deviation of the likelihood-ratio test from the whole protein. B) Alignment of the N-terminus of the Dbp1/Ded1 homologs illustrates the rate heterogeneity amongst columns and highlights the short length of a putative motif (black rectangle zoom). Blue shade represents the percentage identity. C) Alignment of the N-terminus of a simulated protein based on Dbp1/Ded1 using our ‘realistic’ simulation of evolution (see Methods). D) Histogram shows the p-value distribution obtained from set of protein sequences that were evolved as in C). Grey shaded area indicates the expected proportion of tests. Circles indicate the distribution of p-values obtained from the likelihood-ratio test described in A) when the test statistic is assumed to be chi-squared distributed (black circles) or non-central chi-squared distributed (white circles, “corrected”).

We have previously shown that short linear motifs can be predicted based on their conservation relative to their surrounding regions [23]. We sought to detect regulatory divergence in proteins by looking for statistical signals of lineage-specific evolutionary rate changes in predicted short linear motifs in multiple sequence alignments. Likelihood-ratio tests have previously been used to detect differences in rate of evolution of full-length yeast proteins after the whole-genome duplication [16]. We sought to perform essentially the same test to identify short linear motifs whose rate of evolution changed significantly after gene duplication. To do so, we first predicted short linear motifs within proteins of species that have diverged prior to the yeast whole-genome duplication (see Methods) and mapped the location of the predicted short linear motifs to the genes post-duplication (Fig. 1A). Using a likelihood-ratio test [38], we tested whether two rates of evolution (one for the post-duplication clade and one for the remainder of the phylogenetic tree) explain the data significantly better than one single rate of evolution common to the whole tree (see Methods). This test is performed once for genes that reverted to single-copy, and twice in retained duplicates (one for each post-WGD protein).

Previous efforts to identify changes in evolutionary rate have shown that the likelihood-ratio test statistic often deviates from the expected chi-squared null distribution even when there is truly no change in rate of evolution [37], [39]. Indeed, when we performed simulations of molecular evolution with no changes in rate of evolution specific to the short linear motifs (Fig. 1B–C, see Methods), but included realistic aspects of the evolutionary process (such as rate heterogeneity, insertions and deletions, etc.), we found that the likelihood ratio test falsely identified increased rates of evolution after gene duplication (Fig. 1D, black circles, Text S1).

We hypothesized that the increased rate of false rejections was because the additional evolutionary rate parameter in the alternative hypothesis (that is supposed to capture the change in selective constraints) can also model some of the background heterogeneity in evolutionary rate (due to alignment errors, non-stationary and non-homogeneous evolution, etc.).

Under assumptions that 1) the majority of the tests performed are truly null, and that 2) the deviation of the real data from the models assumed by the test is consistent over the columns of the multiple sequence alignment, the distribution of the likelihood-ratio test follows a non-central chi-squared distribution with a data-dependent non-central parameter (see Methods). This non-central parameter (the expected increase in the test statistic from ‘fitting’ some of the heterogeneous background process using the likelihood ratio test) is the product of the Kullback-Leibler (KL) divergence D_KL_, (the “fit” or the expected log-likelihood ratio of the alternative hypothesis over the null hypothesis given the data see Methods) and the number of data points used to compute the likelihood-ratio test. Larger KL divergence means larger deviation of the background distribution from the null model assumed by the test. To use this in practice, we first estimate a non-central parameter using sequence data generated by a background heterogeneous evolution process and then use the non-central chi-squared distribution to obtain p-values for our test (see Methods). Extensive simulations on full length proteins with non-stationary and non-homogeneous evolution, including alignment errors, showed that this approach works as expected and yields uniform p-values (see Text S1).

We applied this approach to our ‘realistic’ simulation (Fig. 1C for an example protein) by calculating a KL divergence parameter for each protein (see Methods) and obtained p-values for each likelihood-ratio test (for each short linear motif) in that protein. This procedure reduced the false-rejection rate (Fig. 1D, white circles) and p-values were nearly uniform.

### Frequent post-duplication changes in constraints in motifs

**Figure 2:**
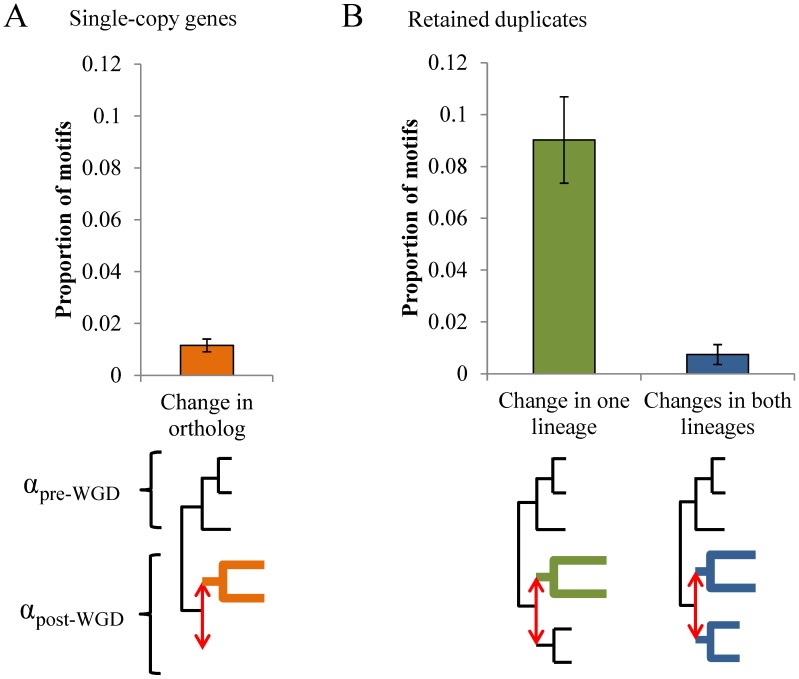
Regulatory turnover after gene duplication. A-B) The proportion of motifs with changes in constraints at a 5% false-discovery rate is significantly larger than in genes with retained duplicates (B) than in single-copy genes (A). Error bars represent the 95% confidence interval of the estimated proportion (binomial distribution). Bolded clades are clades with significant changes in constraints. α is the rate of evolution.

Having confirmed that our approach to detect type I functional divergence could be applied on short linear motifs, we then analyzed our set of protein alignments. After correction for multiple testing, we identified 159 short linear motifs with significantly different rates of evolution after gene duplication at a false discovery rate of 5% (see Methods, S1 Table). This corresponds to 1.2% of the motifs identified in single-copy genes (67/5825 significant motifs, Fig. 2A) and 9.8% of the identified motifs in retained duplicates (92/942 significant motifs, Fig. 2B). Because motifs in retained duplicates are tested twice (once per branch), changes in constraints are approximately 4.5 times more frequent in retained duplicates versus single-copy proteins (5.26% vs 1.15% of LRTs, p-value <10^-20^, Fisher's exact test).

Our previous ‘realistic’ simulation had no intended site-specific changes in constraints. Despite this, our pipeline (including the non-central correction) identified 0.059% of the motifs in simulated single-copy proteins (4/6753 significant motifs) and 0.55% of the motifs in simulated retained paralogs (6/1083 significant motifs) to have significantly different rates of evolution after false-discovery rate correction. Using these values as our estimate of false positives due to possible computational artifacts (such as misalignments) or due to incorrect non-central parameter estimation for the null distribution of the likelihood-ratio test statistic, we expect that 5 motifs in duplicates and 3 motifs in single-copy genes are artifacts. Therefore, although the false positive rate due to artifacts in retained duplicates is significantly higher than in single-copy genes, the increased proportion of motifs identified with changes in constraints in duplicates cannot be explained by these computational artifacts.

As another negative control, we also looked at whether the flanking regions of the putative short linear motifs (five amino acids on each side of the motifs) showed changes in constraints after gene duplication. After correction for multiple testing, only two flanking regions were identified as having significantly different rates of evolution after gene duplication. Given that these identified changes in constraints on the flanking regions are consistent with our false positive rate, this result indicates that the type I functional divergence we identify in predicted short linear motifs is specific to the motifs and not due to some local change in constraint.

Most of the motifs with changes in constraints in duplicates only occurred in one of the two copies (85/92 motifs retained in duplicates), consistent with the idea of sub-/neo-functionalization after gene duplication through posttranslational regulatory changes [8] (Fig. 2B).

### Lineage bias in post-duplication changes in constraints

One hypothesis as to the fate of paralogous proteins is the duplication-degeneration-complementation (DDC) model [2] which explains the preservation of paralogous proteins by the neutral generation of sub-functionalized copies of proteins. Under this hypothesis, one might expect that both paralogous proteins would show signs of relaxed evolution, but that specific functional regions of each protein showing relaxation in selective constraints would be complementary, such that they partition the functional regions in the ancestral protein. We sought to test whether signs of the DDC model could be detected at the posttranslational regulatory level and found 20 paralog pairs where more than one short sequence was detected as having different rate of evolution after gene duplication (see Methods). Of these, seven showed reciprocal changes in constraints on their motifs, which is consistent with degeneration and complementarity at the posttranslational regulatory level as predicted by the DDC model.

**Figure 3:**
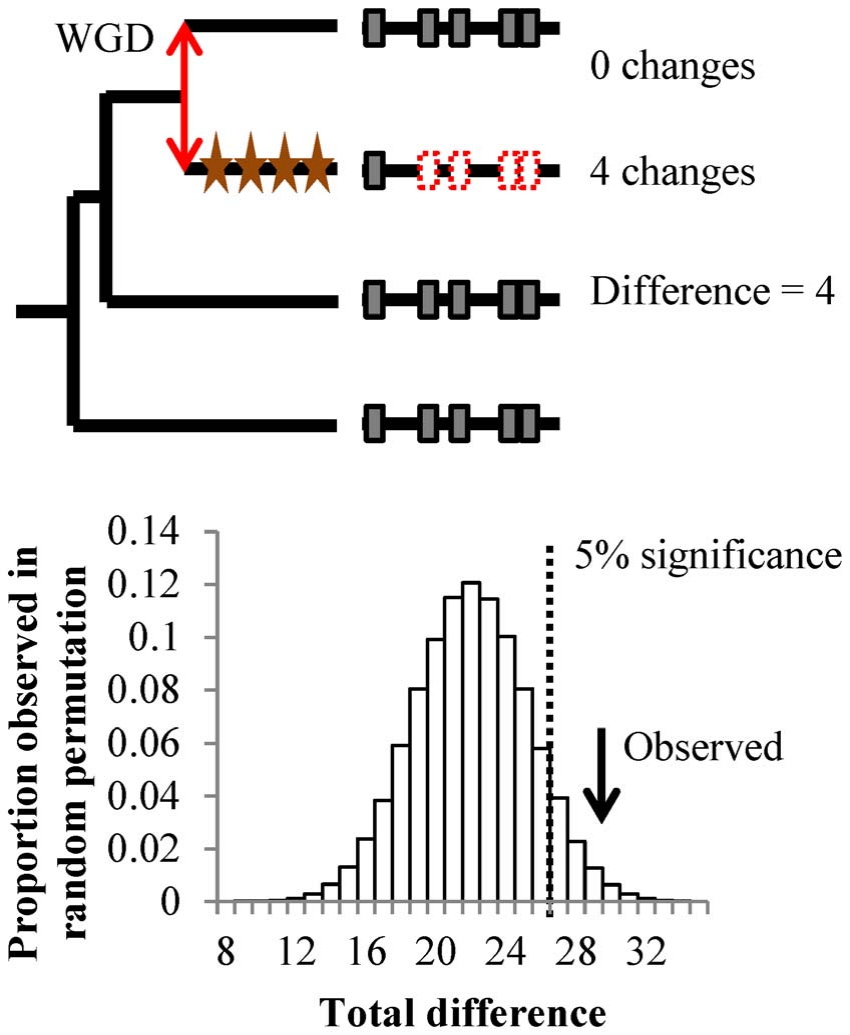
Correlated evolution of short linear motifs. Top panel shows the procedure to obtain the number of lineage specific changes in constraints in a single protein. Red double arrow illustrates the duplication event. Stars represent significant changes in constraints along the lineage. Significant changes in constraints detected on short linear motifs are shown in dotted red boxes. Bottom panel shows the distribution of the total cumulated number of lineage specific changes in constraints from a non-parametric permutation test. Arrow shows the observed total difference for all 20 paralog pairs.

Despite some evidence for complementarity, the majority of paralogs (13/20) with more than a single change in constraints appeared to have a lineage bias in their posttranslational regulatory changes. We tested this using the set of 20 paralog pairs described above and asked whether the motifs were more likely to have correlated evolution than expected by chance. To do so, we randomly permutated the changes in constraints across paralogous pairs to establish the null expectation of random assortment and counted the lineage differences in changes in constraints (see Methods). We ensured that the lineage bias was not caused by technical issues, such as large-scale alignment errors or bipartite motifs being predicted as two motifs by the phylo-HMM, by grouping motifs when they were within 35 amino acids of each other for this test (see Methods). This analysis revealed a lineage bias in changes in constraints for regulatory sequences (p-value  = 0.01106, one-tailed non-parametric permutation test, Fig. 3). Therefore, proteins that change function after duplication may typically change multiple short linear motifs in concert, consistent with the idea that multiple regulatory mechanisms often work together to control protein function. For example, multisite phosphorylation from individual or multiple kinases can form intricate regulatory modules on single proteins (reviewed in [40]) and these clusters of phosphorylation sites have been found to be frequently conserved through evolution [41]–[43] and have been shown to turnover [44].

### Amino acid level resolving power allows detection of additional changes after gene duplication

The increase in resolving power obtained by analysing short linear motifs allowed us to determine whether specific regions within the paralogous proteins differed in their selective constraints. We wanted to test if this amino acid level analysis could also allow us to detect signatures of functional divergence even when the rate of evolution of the whole protein after duplication did not appear to be different than the pre-WGD clade.

Using similar methodologies as previous studies [16], we found that 57% of the paralog pairs showed no evidence of significant increase in rate of evolution of the whole protein in either of the two lineages. This value is slightly higher than that obtained previously (44% [16]), which we attribute to either a different gene set or methodology, or to the non-central correction that we applied. Nevertheless, we then searched within these proteins for motifs with significant changes in constraints. Doing so, we identified 37 motifs in 28 paralogous pairs, and 46 motifs in 43 single-copy proteins. This indicates that an analysis of evolutionary rate differences using higher resolving power of functional sequences within proteins can identify additional sources of functional divergences than analyses at the whole protein level.

### Post-duplication changes in constraints are associated with changes in regulation

If changes in posttranslational regulation are important for functional divergence after gene duplication, we expect the changes in constraints in short linear motifs that we detected to point to functional differences between paralogous proteins. A previous study investigated changes in localization after gene duplication by taking advantage of the systematic green fluorescently-tagged protein collection in budding yeast [7], [45] and categorized paralog pairs as having different or similar subcellular localization. We sought to test if motifs present in paralog pairs with different subcellular localizations were more likely to turnover after gene duplication. Motifs with changes in constraints were more than twice as likely to appear in proteins with detected changes in localization (26/209 motifs with changes in constraints in proteins with different localization vs 12/197 in proteins with similar localization, p-value  = 0.032, permutation test), providing evidence that proteins with changes in localization are more likely to have evolved differences in short linear motifs. We were concerned that this result could be primarily driven by the fact that paralog pairs with changes in localization had significantly higher rates of evolution [7], for example if our non-central correction was not adequate. However, we only found a modest increase in rate of evolution for the paralogs with changes in constraints on motifs and this increase was not significant (two-tailed p-value  = 0.093, Mann-Whitney U test on *Dn* estimated previously [7]). Considering that we have more power to detect changes in constraints in more rapidly evolving proteins, this further suggests that our non-central correction has controlled for the overall protein rate of evolution.

We next tested if the changes in constraints we predicted corresponded to interpretable differences in posttranslational regulation by analyzing experimentally characterized motifs (same set as in [23]) that overlapped with segments predicted to have a change in constraint in paralogous proteins. In addition, we also wanted to confirm that the differences were not specific to *S. cerevisiae* by looking at the presence or absence of motifs in the other species we analyzed.

**Figure 4:**
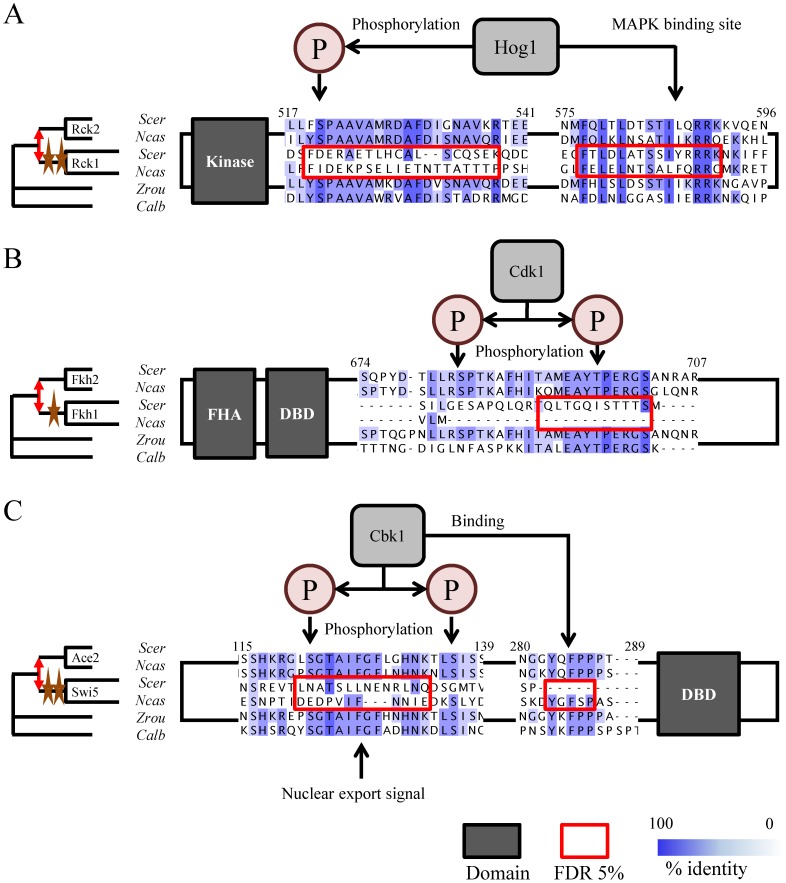
Examples of known regulatory motifs with changes in constraints. Alignment of the short linear motifs with known function (indicated with arrows) and significant changes in constraints (red boxes) after gene duplication from representative species. A) The Rck2 protein is known to bind and be phosphorylated by Hog1 kinase at two motifs that have significant changes in constraints after gene duplication. Numbers indicate residue position within the *S. cerevisiae* Rck2 protein. The two identified motifs occur at aa519-538 and aa577-591 for Rck2, and changed constraints within the aligned region aa439-456 and aa492-506 in Rck1. These overlap with the known phosphorylation site in Rck2 (aa520) and the MAP kinase binding site (aa492-506) in Rck2. Both Rck2 and Rck1 retain kinase function. B) The Fkh2 protein is known to be phosphorylated by Cdk1 at two phosphorylation sites on a region shown to have significant changes in constraints after gene duplication. Numbers indicate residue position within the *S. cerevisiae* Fkh2 protein. The identified motif occurs at region aa692-702 in Fkh2 and has changed constraint in the aligned region aa459-469 in Fkh1. One of the known phosphorylation site in Fkh2 occurs within this region at aa697. Fkh2 and Fkh1 retain their forkhead-associated domain (FHA) and DNA binding domain (DBD). C) The Ace2 protein is known to bind and be phosphorylated by Cbk1 kinase at two motifs that have significant changes in constraints after gene duplication. Numbers indicate residue position within the *S. cerevisiae* Ace2 protein. The two identified motifs occur at aa121-134 and aa283-287 in Ace2, and changed constraints within the aligned region aa115-128 and aa247-248 (it is a gap) in Swi5. These overlap with the known phosphorylation site in Ace2 (aa122) and the Cbk1 binding site (aa283-286) in Ace2. Both Ace2 and Swi5 retain their DNA binding domain (DBD). Stars represent significant changes in constraints along the lineage. Red double arrow illustrates the duplication event. aa: amino acid position. Scer: *S. cerevisiae*, Ncas: *N. castellii*, Zrou: *Z. rouxii*, Calb: *C. albicans*.

Of these, the paralog pair Rck1/Rck2 contained two predicted motifs that were found to have significant changes in constraints in the Rck1 protein. Interestingly, both motifs are involved in Hog1 signaling [46], [47]. Consistent with our predictions, Rck2 is known to be regulated by Hog1, while Rck1 is thought not to be regulated by Hog1 [47]. However, while our algorithm identified that the motif required for Hog1 binding in Rck2 was evolving more rapidly in Rck1, it is clear that Rck1 preserved some of the critical residues required for binding to Hog1, yet its binding activity to Hog1 has been shown to be poor [47]. This suggests that: 1) the protein ancestral to Rck1/Rck2 is likely to also be regulated by Hog1, and 2) that Rck1 is likely to be regulated in a different manner, having lost or changed critical regulatory sequences after the duplication event (Fig. 4A).

Another clear example where experimentally characterized regulation of one paralog appears to have been lost in the other following gene duplication is in the Fkh2/Fkh1 paralogous pair of transcription factors. While both proteins play a role in cell-cycle progression, they are known to have non-redundant functions [48]. For example, Fkh2, but not Fkh1, associates with Mcm1 [49]. Another important function of the Fkh2 protein that is absent in Fkh1 is its ability to recruit the transcriptional co-activator Ndd1. This interaction is mediated by at least two adjacent Cdk1 phosphorylation sites [50], one of which is found to have significant changes in constraints in the Fkh1 lineage. The other phosphorylation site is not predicted by our motif prediction algorithm but is also likely to have changed constraints. We speculate that the ancestral protein to Fkh1/Fkh2 may also have bound Ndd1 in a Cdk1-dependent manner, but Fkh1's regulation appears to have changed, possibly to accommodate new functional roles (Fig. 4B).

A third example could be found in the Ace2/Swi5 paralog pair, important cell-cycle regulated proteins known to localize differently in budding yeast [51]. These two proteins have been extensively characterized, with several major posttranslational regulatory sequences identified [52], [53]. Two of these have significant p-values in our analysis, suggesting that changes in constraints occurred within the Swi5 lineage. One of these is the Cbk1-regulated nuclear export signal, known to give Ace2 its daughter-cell specific nuclear localization [52], and another is a putative Cbk1-binding motif [23] (Fig. 4C). In Ace2, Cbk1 phosphorylation prevents nuclear export and Cbk1 is only active in daughter cells [52]. Therefore, we hypothesize that the ancestral protein to the Ace2/Swi5 paralog pair was also regulated by Cbk1 to provide daughter-cell specific nuclear localization, but that loss of these important signals allowed Swi5 to localize to both mother and daughter cells' nuclei.

### Pre-WGD Ace2 localizes asymmetrically

**Figure 5:**
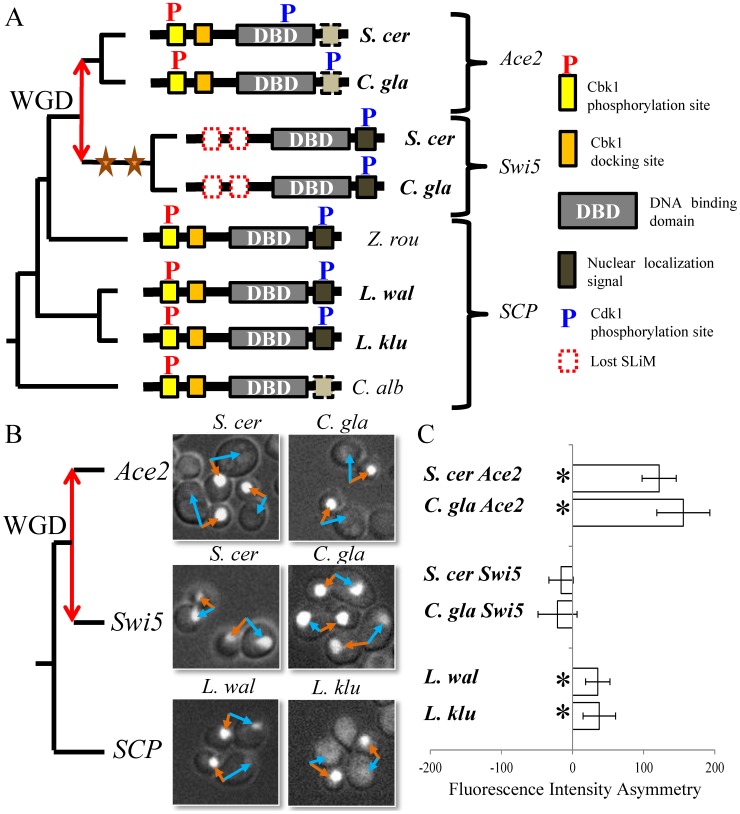
Posttranslational change in regulation after gene duplication in Swi5 and Ace2. A) Schematic of the gene tree relating the Ace2/Swi5 paralog pair with diagram of protein features found in proteins from different yeast species. Bolded species name indicate cloned genes assayed for localization in *S. cerevisiae*. The nuclear localization signal characterized in Swi5 is putatively altered and may not be functionally homologous in *Candida* and Ace2, but this difference was not predicted in our analysis (see Discussion and S3 Figure). B) Green-fluorescent protein tagged genes cloned from the labeled species were assayed for their localization in unsynchronized *S. cerevisiae* cells. Two representatives of each pre-/post-WGD genes were assayed. Orange and blue arrows indicate representative bud and mother nucleus pairs. C) The fluorescence intensity of the nucleus in cells expressing the labeled proteins was quantified, and mean difference of the intensity (bud-mother) is used as the measure of asymmetry (unfilled bars). Error bars show 95% confidence interval of the mean. Stars indicate 5% statistical significance. Red double arrow illustrates the duplication event. Scer: *S. cerevisiae*, Cgla: *C. glabrata*, Zrou: *Z. rouxii*, Lwal: *L. waltii*, Lklu: *L. kluyveri*, Calb: *C. albicans*.

To confirm our sequence-based predictions about evolutionary divergence, we focused on the Swi5/Ace2 paralog pair. It has previously been shown that these motifs in the extant *S. cerevisiae* proteins control the differential localization pattern of the paralogs [52]. Because the ancestral protein likely contained critical regulatory motifs, we hypothesized that it was also regulated by Cbk1, and localized asymmetrically in the daughter cell (Fig. 4C). We therefore wanted to assess whether the localization before and after the gene duplication was consistent with our sequence analysis. To test this, we cloned and replaced the *S. cerevisiae* endogenous *SWI5* gene with GFP-tagged Swi5/Ace2 homologs from multiple species that diverged before and after the whole-genome duplication and quantitatively assayed their localization pattern using fluorescence microscopy (Fig. 5A, see Methods and Text S1).

Upon visual inspection, consistent with our predictions, both single-copy genes localized in an Ace2-like pattern with clear daughter specific localization (Fig. 5B). To quantitatively compare the localization asymmetry of the retained duplicates and the single-copy proteins, we manually quantified the nuclear fluorescence (see Methods) and computed the difference between fluorescence intensity in bud and mother cells, and used this as measure of asymmetry. While we could not reject the null hypothesis of symmetry in bud and mother cell localization for Swi5, the single-copy proteins and Ace2 showed statistically significant asymmetry, consistent with our visual inspections (Fig. 5C, p-value <0.05). The most parsimonious explanation for these results is that the ancestral protein also showed asymmetrical nuclear localization.

Interestingly, we noted that the quantitative measure of asymmetry for the single-copy proteins was not as extreme as the post-duplicate Ace2 (Fig. 5C). We also observed several cells with clear mother cell GFP localization just as observed for Swi5 (e.g., Fig. 5B, L. wal panel, top cell, blue arrow). This suggests that the single-copy genes may actually represent a mixture of the Ace2 and Swi5 localization patterns, and may be more consistent with sub-functionalization of the ancestral function, as opposed to the simple lineage specific losses predicted based on sequence analysis alone (see Discussion).

To confirm our prediction that the changes in regulation were not specific to the *S. cerevisiae* lineage and occurred during the period of rapid diversification immediately following the whole-genome duplication, we also examined the corresponding genes from *C. glabrata* (a budding yeast species that diverged from *S. cerevisiae* after the whole genome duplication) and found similar patterns of localization to *S. cerevisiae*. This supports our prediction that the change in localization in the two paralogs most likely occurred shortly after the gene duplication event (Fig. 5B,C) and rules out the possibility that the changes we observe are simply due to a problem with expressing foreign proteins in *S. cerevisiae*. Although we cannot rule out more complicated artifacts due to the expression of heterologous proteins, because we observe consistent localization in two proteins that diverged before and two proteins that diverged after the gene duplication, we consider such artifacts unlikely.

Although our results only provide indirect evidence for the role of the motifs in the localization of the heterologous proteins we tested, we believe that, along with the experimental evidence for the mutations on the motifs that was performed previously by [52], that these experiments support our prediction that the asymmetric localization pattern of Ace2 was present in the single-copy ancestral protein, and this asymmetry was lost after the gene duplication in Swi5 due to losses of specific posttranslational regulatory sequences.

## Discussion

In this study, we have analyzed the evolution of short linear motifs in protein disordered regions after gene duplication and found that regulatory change is likely to contribute to functional divergence in paralogous genes. An important outstanding question in this analysis is whether the functional changes we identify are adaptive. Previous studies have shown adaptation due to specific changes in posttranslational regulation [54], however general molecular mechanisms for these adaptive posttranslational regulatory changes are still under study. The resolution of adaptive conflicts has been suggested as a model for adaptation of paralogous copies of multifunctional genes after duplication [4] and differential patterns of posttranslational regulation could be an example of resolved ‘multifunctionality’. For example, in our analysis of the Ace2 and Swi5 paralogous pair, we observed that the asymmetry of the single-copy proteins was reduced when compared to the post-duplicate Ace2 (Fig. 5C). Although we cannot rule out that these single-copy proteins have other mechanisms within these species that confer daughter specific localization (as we use a heterologous system to test for their localization), we believe that this observation may instead be due to a Swi5-specific motif. Indeed, the characterized nuclear localization signal (NLS) of Swi5 [55] was not predicted in our analysis, most likely due to its proximity to the DNA-binding domain, or to the weak conservation of the residues associated with the NLS in the *Candida* species. This NLS of 20 amino acids spans 50 alignment columns within our alignment, and upon close inspection appears to show that the single-copy protein contains high sequence similarity to the Swi5 NLS and that the Ace2 protein and proteins from *Candida* have a more dissimilar one, suggesting that they might not be functionally homologous (S3 Figure). This hypothesis is consistent with the predominantly Ace2-like localization pattern of the orthologous protein in the *Candida* clade [56]. We speculate that this NLS is responsible for the Swi5-like pattern of localization in both Swi5 and the single-copy protein. Given that Swi5 is known to enter the nucleus slightly before Ace2 and becomes degraded before Ace2 exits the daughter-cell nucleus [51], [57], the observed pattern for the single-copy protein is consistent with first localizing to both mother and bud nucleus as Swi5, and subsequent nuclear export from the mother cell. We propose that the differential localization pattern of the Ace2/Swi5 paralogs is a repartitioning of localization of the ancestral protein due to sub-functionalization of the short linear motifs present in the ancestral protein.

In this study, we have identified several putative motifs that have changed constraints within proteins after the whole-genome duplication in budding yeasts. Our methodology to identify changes in evolutionary rate in very small motifs relies on a correction to the distribution of the likelihood-ratio test statistic to control for possible ‘protein level’ background heterogeneous evolution that can be encountered. These ‘protein level’ effects, such as changes in protein expression levels [58] and divergence due to changes in essentiality or gene function [59], [60], have been shown to be major issues in evaluating correlated changes in evolutionary rates between interacting proteins [61], [62]. These effects are likely to be encountered in our set of paralogous proteins. Therefore, we ensured that the identification of divergent short linear motifs is unlikely to be caused by these “protein level” effects by correcting the null distribution of the likelihood-ratio test to take account of the whole protein's deviation to the null model assumed by the test. Other methodologies have been previously proposed to empirically obtain the distribution of the likelihood-ratio test statistic [37]. Our approach is similar; however we only estimate one parameter (the non-central parameter) because in our case it sufficiently describes the null distribution. Both approaches (empirically-derived null distribution and estimation of the non-central parameter) have the caveat that they rely on having several data points (in our case alignment columns) that are assumed to be null distributed. An additional constraint of our approach is that it requires that the null distributed data evolves under a shared and constant background heterogeneous evolutionary process to obtain the KL divergence. Therefore, it cannot accurately produce an adequate null distribution under cases where recombination has occurred in a gene, for example. Nevertheless, this approach can be simpler and faster than the permutation tests when performed on genome-wide data where we expect a small proportion of tests to reveal functional divergence. We believe that the non-central chi-squared null-distribution can be applied to other important tests in molecular evolution where genome-scale data are available and where the assumptions of the chi-squared distribution of the likelihood-ratio test statistic are violated; however this is still under study.

Our study on short linear motifs reveals that posttranslational regulatory evolution is widespread after gene duplication. However, an important limitation of our study is that it cannot identify novel regulatory sequences that have appeared along any lineage or that occur within structured regions, in part due to the way motifs are predicted. Additional genomic sequences such as population data or from additional post-WGD species may allow further analyses of functional changes in the budding yeast after gene-duplication. These types of analyses are likely to uncover even more functional variations between paralogous proteins than were suggested by protein-wide and motif-wide analyses.

Nevertheless, our results are consistent with several results suggested by other studies [8], [9]: posttranslational regulatory change may underlie an important number of observed functional differences between paralogous proteins. This appears analogous to the models of functional divergence after gene duplication due to transcriptional regulatory change [2]. These parallels between transcriptional and post-translational regulatory evolution [29] suggest that transcription factor binding sites in non-coding DNA are analogous to SLiMs in proteins. In the former, the rapid transcriptional regulatory evolution is facilitated by the rapid evolution and lack of strong constraints on non-coding DNA. In the case of post-translational regulatory evolution, because SLiMs are typically found in protein disordered regions which evolve rapidly due to lack of structural constraints, changes in motifs in disordered regions may be a general means to facilitate functional evolution [63].

## Methods

### Alignment of related species of yeasts

We based the orthology assignment on the data from the Fungal Orthogroups Repository [64] because it contained both sequences from Candida species and budding yeasts. Protein sequences and orthology assignment from six Candida yeast species [*Candida tropicalis*, *Candida albicans*, *Candida parapsilosis*, *Candida lusitaniae*, *Candida guilliermondii*, *Debaryomyces hansenii*] were obtained from the Fungal Orthogroups Repository. When several protein sequences from the Fungal Orthogroups Repository were mapped to a single budding yeast orthology group, only the most similar sequence as assessed by blast scores was chosen. The six Candida genes, along with the *Saccharomyces cerevisiae* gene, were supplemented with protein sequences and orthology assignment from 19 additional related budding yeast species [*Saccharomyces mikatae*, *Saccharomyces bayanus var. uvarum*, *Saccharomyces kudriavzevii*, *Candida glabrata*, *Kazachstania Africana*, *Kazachstania naganishii*, *Naumovozyma castellii*, *Naumovozyma dairenensis*, *Tetrapisispora blattae*, *Tetrapisispora phaffii*, *Vanderwaltozyma polyspora*, *Zygosaccharomyces rouxii*, *Torulaspora delbrueckii*, *Kluyveroymces lactis*, *Eremothecium gossypii*, *Eremothecium cymbalariae*, *Lachancea kluyveri*, *Lachancea thermotolerans*, *Lachancea waltii*] that were obtained from the Yeast Gene Order Browser [65]. By basing our orthology assignment on the species that have not undergone a whole-genome duplication, our single-copy genes do not include singletons (newly arisen genes after the whole-genome duplication), and our set of retained duplicates do not include small-scale duplicates (duplications that arose after the whole-genome duplication). In total, 452 alignments of retained duplicates and 3566 alignments of single-copy proteins were used in our analysis.

Protein sequences were then aligned using MAFFT v6.864b with the —auto flag at default settings [66].

### Conserved segment prediction

We sought to predict small functional regions that could be labeled as short linear motifs. Because we were interested in functional segments that could be identified before the whole-genome duplication [67], we first removed from the multiple sequence alignment the sets of proteins from species that had undergone the whole-genome duplication and predicted short linear motifs within the remaining species (which we refer to as the ‘pre-WGD clade’). To identify short linear motifs, we used a phylogenetic hidden Markov model (phylo-HMM) [23]. Briefly, this method identifies highly conserved short amino acid sequences within disordered regions of proteins. The unstructured regions are predicted by DISOPRED2 [68], filtered for coiled coils using pFilt [69] and for repetitive regions using the SEG algorithm [70]. We also use the phylo-HMM to filter out large conserved regions as we consider them likely to be structural regions. In a previous study, the phylo-HMM approach identified 104 of 352 known motifs with a false positive rate of 1 in 9000 amino acids [23].

In addition to the heuristics used in [23], we now also assume that a scaling factor of rates of evolution within the conserved state is sampled from a discretized Gamma distribution with eight categories [71] with a fixed alpha and beta parameter of 0.6, which was chosen as a heuristic that allowed predictions of large conserved regions (>35aa) interspersed by a few fast evolving columns. We now obtain the rates of evolution through a Newton-Raphson procedure, and used a window size of 31 alignment columns for the calculation of the background rate.

Because the phylo-HMM tends to classify single insertion/deletion events as slow evolving regions, motifs are trimmed on either end to remove regions that are over 50% gaps or are filtered out if the prediction itself contains over 50% gaps.

Flanking regions of the predicted conserved segments consisted of five alignment columns on each side.

### Likelihood-ratio test of multiple rates of evolution

We sought to systematically identify short linear motifs that evolve at a different rate after the whole-genome duplication. To do so, each predicted motif from the pre-WGD clade was mapped back into the complete alignment.

Each predicted motif was then analyzed using the PAML package [72] by a likelihood-ratio test that compares the null hypothesis (H_0_) that motifs before and after the whole-genome duplication are evolving at the same rate, to a model (H_1_) with two distinct rates [38] (PAML program: AAML, clock = 2, cleandata = 0, fix_omega = 0, ncatG = 8). Likelihood-ratio tests have been previously used to study the evolution of the yeast paralogs generated in the WGD [16]. Our test differs from this previous application of the likelihood-ratio test, because we compared the evolutionary rate on each paralogous clade (post-WGD_1 and post-WGD_2) to the evolutionary rate on the lineages that diverged before the whole-genome duplication (pre-WGD) one at a time. Formally, the likelihood-ratio test (LRT) is:
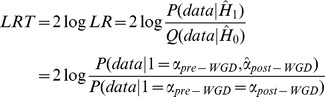
where the data corresponds to the motif segment within the multiple sequence alignment, and α_clade_ represents the rate for corresponding clades. In this model [38], α is a scaling factor by which the estimated branch lengths are multiplied, and one of the rates always defaults to 1. Therefore, under the null hypothesis H_0_, the single rate is equal to 1, while the alternative hypothesis H_1_ allows one of the two rates to be different than 1 and it is estimated by maximum likelihood. Because these models are nested, under the null hypothesis H_0_, the distribution of the likelihood-ratio test statistic (LRT) follows the chi-squared distribution with degrees of freedom equal to 1 [73] (see the next Methods section for the correction to the chi-squared distribution performed when assumptions of the test are violated). Although it is in principle possible using this test to find short linear motifs that evolve either slower or faster than the proteins in which they are found, because short linear motifs are predicted on the basis of their conservation in the pre-WGD clade, we only expect to identify motifs with faster rates of evolution after the whole-genome duplication.

We estimated the false discovery rate using a slight modification of the procedure described in [74] to obtain a threshold for significant p-values. We modified this approach because when applying the LRT described above to our alignments of the yeast proteome, we observed a large number of tests resulting in LRTs of exactly zero (thus having a p-value of 1, e.g. Fig. 1D), many of which correspond to motifs where no information can be inferred about their rate of evolution. For example, in our real data, for 284/498 of these LRTs of exactly zero, we observed no amino acid differences in the multiple alignments and therefore have no power to estimate a change in evolutionary rate. Because we observed that p-values between 0.6 and 0.95 appeared uniform as expected for the distribution of truly null p-values, we used this range only to estimate the false discovery rate (FDR). We counted 1836 p-values between 0.6 and 0.95 out of a total of 7709 tests. If we assume that all these p-values correspond to truly null hypotheses, then we can estimate the proportion of null tests (π_0_) by 1836/(7709*(0.95-0.6))  = 0.6804. The FDR at p-value threshold *t* is therefore estimated as:
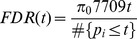



We considered p-values as significant where this FDR is lower than 0.05.

### Correction for data heterogeneity due to violations of model assumptions about protein evolution

Increased evolutionary rate after gene duplication is frequently observed in entire proteins [17]. We reasoned that short linear motifs within these proteins may also show the same changes in protein-level selective constraints. Furthermore, because mutations may not be homogeneous over the phylogeny (e.g., due to lineage specific changes in GC content), proteins might show biases in their substitution process that are not accounted for by the models assumed in the LRT. Because we were interested in short linear motif evolution, we wished to test for *additional* changes in motifs using the heterogeneity of protein evolution as the “background”. In this case, we can still compute the LRT statistic, but the test statistic no longer follows the standard chi-squared null distribution because the heterogeneity in rates and patterns of protein evolution can be ‘fit’ using the additional parameter in the alternative hypothesis. This biases the test to reject the null hypothesis and leads to detection of false positives. A permutation test has been proposed for this case [37] however, in our case, this test must be performed for each individual predicted motif, and these permutation tests may lack power for genome-wide analyses. We therefore devised another strategy by which we can approximate the distribution of the LRT statistic under a heterogeneous background process in protein evolution.

We assume that evolution of each alignment column is independent and is possibly evolving under a heterogeneous background process after the whole genome duplication event. This heterogeneity that affects the whole protein could be due, for example, to changes in expression level, lineage-specific changes in GC content or alignment errors. The likelihood of the data generated under this scenario can be computed under the alternative hypothesis H_1_ where there has been a change in constraints P(data|H_1_), or under the ‘null hypothesis’ where evolutionary rate has remained constant, Q(data|H_0_). We note that H_1_ can capture only some of the true heterogeneity in the data using the additional rate parameter, and the null model H_0_ captures even less. If θ is a parameter space and β the possible values of those parameters, then there may exist sets of values β* in the parameter space of the alternative hypothesis θ_H1_ that captures some of this heterogeneity and that cannot be captured by the values β_0_ in the parameter space of the null hypothesis (θ_H0_). Although this heterogeneous background process does not produce data following a generative process with parameters and values β*, we only seek the extra ‘fit’ obtained from the parameter space θ_H1_ that cannot be captured by the parameter space θ_H0_.

This fit can be summarized by the expectation of the log-likelihood-ratio of the two models, where the expectation is taken using the probabilities P, which is the Kullback-Leibler (KL) divergence D_KL_(P||Q). This measures the additional amount of deviation of the possibly heterogeneous background captured by the alternative hypothesis relative to the null hypothesis.
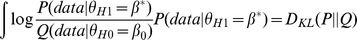



In practice, we cannot necessarily parameterize the heterogeneity in the background evolutionary process, for example if it is due to alignment errors (i.e. it is difficult to estimate β* or how data is generated from this heterogeneous process). Nevertheless, the distribution of the likelihood-ratio test statistic (LRT) when we test the alternative hypothesis H_1_ vs H_0_ (by maximizing the ‘fit’), is related to the KL divergence as follows. Given that the data used to compute the LRT are truly drawn from P, the distribution of the likelihood-ratio test statistic converges to a data-dependent *non-central* chi-squared distribution, χ^2^(*k*,*λ*), parametrized by the “non-centrality parameter” *λ* and the degrees of freedom *k*. The non-centrality parameter is given by *λ* = 2 *L D_KL_(P||Q)*, where *L* is the number of data points used in the LRT [75]. To estimate D_KL_(P||Q), we note that the mean of the LRT when data is drawn from P must be equal to the mean of the non-central chi-squared, which is given by *k*+*λ*. Therefore,
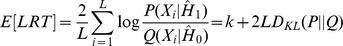
where X_i_ is the data at an alignment column i, *k* is 1 in our case and *L* in our case is the number of alignment columns.

Under the assumption of independence between alignment columns, D_KL_ can be estimated from the whole alignment using a single likelihood-ratio test, which we believe is reliable since *L* is the number of alignment columns in the whole protein and is typically large, and we assume that the background process operates uniformly over the alignment columns. Therefore, we let 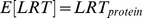 and use: 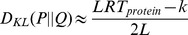



We note that because the motif is small in comparison to the whole protein (which we use to estimate P), its contribution to the calculation of D_KL_ is small and unlikely to affect the results.

While the expectation of the likelihood-ratio test statistic (E[LRT]) is always greater or equal to the degrees of freedom *k*, the obtained likelihood-ratio test statistic for a single protein LRT_protein_ may be smaller than *k*, especially when D_KL_ is small. In these cases, we assume that D_KL_ is equal to the parameter estimated for proteome-wide (species) evolution (see below).

We note that P = Q implies β* = β_0_, which indicates that the data has no source of background heterogeneity that is better captured by the alternative hypothesis than by the null hypothesis. In that case, D_KL_ is zero and this approach simplifies to the standard chi-squared distribution. Further, although it is possible to formulate a likelihood-ratio test with estimated β* as the values of the parameters of the null hypothesis (akin to modeling more complex evolutionary processes in the test), there are several advantages of modeling the extra ‘fit’ instead. First, it is a single value, and second, it is directional (such that rejection of the null hypothesis occurs when values of the parameters are farther from β_0_ than from β*).

This estimate of the non-centrality parameter gives us a new null distribution for the LRT statistic for the predicted motifs in each protein. Since these motifs are short segments chosen from the entire alignment, we can compute the probability of having observed an LRT statistic as extreme (or more) in a short segment, given the length of the motif and the null distribution estimate for that protein. Therefore, the p-value for each motif, *m*, is given by the non-central chi-squared with 1 degree of freedom and non-centrality *λ_m_*.
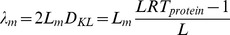
where *L_m_* is the length of the short linear motif. A closed-form solution exists, which we used, for the cumulative distribution of the non-central chi-squared with one degree of freedom:
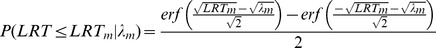



Where erf is the error function, *LRT_m_* is the LRT statistic computed (by PAML) for the motif *m*, and *λ_m_* is as above. In more general cases (i.e. *k*>1), this computation can be performed using several algorithms (see e.g. [76]).

We also noticed that the species used in our study appeared to evolve in a manner that differed from the single rate of evolution null hypothesis (H_0_), even for single-copy proteins. To correct for this additional source of heterogeneity, we estimated another D_KL_ parameter using the whole proteome to rule out any effect on the short linear motifs that could be explained simply by species-level evolution. This D_KL_ parameter was estimated to be 0.014552523. We therefore obtained two D_KL_ parameters for each motif, and because we wanted to correct for rate differences which could be explained by genome-wide deviation or the individual protein's deviation, we chose the larger parameter while computing the p-values. This chooses the larger p-value, for which we believe no additional multiple-testing correction needs to be performed (in that we believe we are still performing only one test per motif) and allows us to perform a likelihood-ratio test using the standard tools for molecular clock hypothesis testing. Importantly, this global correction means our p-values are always more conservative than the significance values obtained using the standard central chi-squared distribution.

### Simulation of protein evolution

To simulate more ‘realistic’ protein evolution (Fig. 1), we use a similar simulation program as in [23]. We evolve sequences to closely mirror our protein alignments by using every protein in our analysis as a template for a simulated protein. First, AAML is used on every protein alignment to obtain protein-specific branch lengths for the phylogenetic tree (we use the species tree for all proteins). The root sequence is one of the sequences of the alignment (we chose the protein sequence of median length), and a site-specific rate of evolution for each amino acid is inferred by the phylogenetic hidden Markov model, which we use as a scaling factor to evolve the root according to the branch lengths obtained by AAML. Indels are generated as in [23] but site specific rates are propagated to indels, such that insertions have the same rate of evolution as the amino acid positions that created it. To ensure that the sequences were as realistic as possible, we also use two amino acid substitution models: one for ordered regions, and one for disordered regions. These two models differ by their equilibrium, or stationary frequencies, of the 20 amino acids, which is estimated based on DISOPRED2 predictions on the *S. cerevisiae* proteome. The exchangeabilities of amino acid pairs was estimated as a whole on closely related species as in [23]. Because the rate matrix is a product of the stationary frequencies with the exchangeabilities of amino acids [77], the substitution matrix for disordered and ordered regions will tend to create amino acids found in disordered and ordered regions, respectively. These stationary frequencies of amino acids are also used in the production of insertions.

We assigned ordered or disordered regions in the root sequence, and propagated them across the phylogenetic tree. Finally, to ensure that some motifs can be predicted, we do not allow indels within regions that have been predicted as motifs in the ancestor. Our simulated proteins are then evolved according to estimated phylogenetic trees with two different substitution processes (and therefore two different stationary frequencies of amino acids), and with indels. Importantly, we do not include any site specific changes in constraints. After alignment by MAFFT, the full pipeline used to predict short linear motifs and calculate the likelihood-ratio test is then used on the full set of simulated proteins. In principle, none of the motifs are intended to have lineage-specific changes in constraints. However, in practice, computational artifacts may occur during the simulation (such as misalignments, deletions of motifs within a clade, mispredictions of short linear motifs) and these can cause signatures of type I functional divergence. Deletions causing a motif to be removed in one of the lineage are computational artifacts of the simulation because they are unintended; however they also would represent genuine changes in constraints on the motif. However, misalignments and mispredictions of short linear motifs are actual computational artifacts that can also occur within our data. Using this set of simulated proteins, it is therefore possible to conservatively assess how many of the predicted changes in constraint can be explained by these computational artifacts or by incorrect non-central parameter estimation for the null distribution of the likelihood-ratio test statistic.

### Test of correlated evolution

We define correlated evolution to be a tendency for changes in constraints on several functional sequences to occur within only one of the two paralogous proteins. Our test for correlated evolution cumulated the number of conserved segments with changes in constraints within each of the paralogs and asked whether the changes occurred more in a particular direction than expected by chance. Under the null hypothesis, the expected difference in the number of motifs changing in one direction minus the other on one protein should be zero. The sum of all the differences is used as the final test statistic, for which a p-value was obtained by a non-parametric permutation test.

To correct for the possibility that the phylo-HMM mistakenly separated a functional fragment as two motifs due to rapid evolution between the regions, we counted multiple motifs that were close to each other (within 35aa) and that had accelerated evolution on the same lineage as a single motif for the purpose of this test.

### Localization analysis

We wished to test that the localization of Ace2/Swi5 homologous proteins differed by quantifying the intensity of the green fluorescent protein with respect to bud or mother nuclei. We chose to quantify solely the nuclear intensity as these proteins are transcription factors known to shuttle to the nucleus during the cell cycle, and show distinct patterns of nuclear localization [51]. To obtain normalized fluorescence intensity, images were analyzed by manually quantifying the cell and nuclear median green fluorescence. Cell size in pixel count was also quantified in this manner and was used to identify the daughter cells. The difference in fluorescence intensity between the bud and mother cell was used as the index of asymmetry. Cells where the median fluorescence intensity observed was over 240 were discarded as they were potentially too saturated to obtain reliable measures. Statistical significance was calculated using a Z-test.

To determine statistical significance when testing for association between changes in constraints and localization differences as determined by [7], we asked whether the observed fold increase in rate of motif changes in constraints was higher than random permutations of the ‘different’ and ‘similar’ labels of localization.

### Software availability

The updated phylo-HMM and simulation programs can be found at http://www.moseslab.csb.utoronto.ca/phylo_HMM/data.php

## Supporting Information

Text S1 **Supplementary results and methods.**
(PDF)

Figure S1 **The chi-squared approximation of the distribution of likelihood-ratio test on short sequences is conservative**. A) Short amino acid sequences of various lengths were evolved under the WAG model with the same phylogenetic tree that follows a global clock (corresponding to the null model assumed by the test). Grey bars show the expected distribution of p-values if the chi-squared approximation is correct. Data points are the obtained distribution of p-values. B) Short linear motifs of length 7 were evolved using the same procedure as in A) but the phylogenetic tree was scaled to allow for more substitutions per sites, showing that more substitutions do not lead to more false rejections than expected for short sequences.
(TIF)

Figure S2 **P-value distribution of the likelihood-ratio test obtained from chi-squared and non-central chi-squared on simulated data.** A) Amino acid sequences were evolved under the WAG model with or without indels. Grey bars show the distribution of p-values obtained from the likelihood-ratio test when the data are generated according to the model assumed by the test. Circles indicate the distribution of p-values when indels are also included and data is aligned, and the test statistic is assumed to be chi-squared distributed (black circles) or non-central chi-squared distributed (white circles, “corrected”). B) Protein coding DNA sequences were evolved. Grey bars show the distribution of p-values when sequences are evolved under a homogenous and stationary codon frequency model assumed by the test. Circles indicate the distribution of p-values when the model is non-homogenous and the test statistic is assumed to be chi-squared distributed (black circles) or non-central chi-squared distributed (white circles, “corrected”). Squares indicate the distribution of p-values when the indels are also included and the test statistic is assumed to be chi-squared distributed (black squares) or non-central chi-squared distributed (white squares, “corrected”).
(TIF)

Figure S3 **Alignment of the characterized nuclear localization signal of Swi5 and other species.** Green box indicates the characterized nuclear localization signal in the Swi5 lineage. Coordinates of Ace2 are indicated in the alignment. Bolded species indicate species that we tested for their localization in Fig. 5.
(TIF)

Table S1 **Motifs identified and the associated p-value for the changes in constraints.**
(XLSX)

Table S2 **Strains and primer sequences used in our study.**
(XLSX)
