# Supplementary material for: Detecting Functional Divergence after Gene Duplication through Evolutionary Changes in Posttranslational Regulatory Sequences
Source: PLoS Comput Biol. 2014 Dec 4;10(12):e1003977. doi: 10.1371/journal.pcbi.1003977 (PMC4256066; doi:10.1371/journal.pcbi.1003977)
Supplement: Text S1 — Supplementary results and methods. (PDF) [file pcbi.1003977.s001.pdf]

# Text S1. Supplementary results and methods

## Behavior of the likelihood-ratio test on short sequences under the null models

We were first concerned by the fact that these motifs are short, which may prevent the use of the chi-squared approximation for the distribution of the likelihood-ratio test statistics (see main text reference [1]). We evolved sequences of different lengths according to the null model assumed by the test (see Methods) and assessed whether the likelihood-ratio test statistics were chi-squared distributed. If the likelihood-ratio test statistics were chi-squared distributed, then we would expect uniformly distributed p-values. In our simulation of short sequence evolution, we observed that the p-values obtained were not uniform and that there were much fewer rejections of the null hypothesis than expected (Figure S1A). This indicates that this test has low power for very short sequences and that assuming chi-square distribution for the likelihood-ratio test statistic will be conservative. Increasing the branch lengths to allow for more substitutions improved the uniformity slightly, but we never observed higher rates of rejection of the null hypothesis than expected (Figure S1B). In phylogenetics studies involving proteome-wide analyses, increased acceptance of the null hypothesis has been suggested as an acceptable compromise to decrease the levels of false positives that may arise due to invalid model assumptions [1,2]. See the main text for the behavior of the test on more 'realistic' simulations.

## Simulation of protein evolution under various evolutionary models

To illustrate the use of this non-central correction to eliminate false rejections of the null hypothesis when the background evolutionary process deviates from the model assumed by the test, we performed extensive simulation of sequence evolution where there were truly no changes in constraints after gene duplication, but where the simulation violated the model assumed by the

test. In each case, we used a likelihood-ratio test to test whether the data is better explained by two rates of evolution as opposed to a single rate of evolution (using AAML, see reference [38] from the main text, and see Methods). Under the null hypothesis, p-values show a uniform distribution and we use this as a measure for deviations of our test statistic under various simulations. For example, if the p-values are uniform in a simulation with no true positives, then we infer that the null distribution used is correct.

We first simulated proteins evolving under the WAG model (see reference [77] in the main text) according to a single rate of evolution, which is the model assumed by the test, and tested for changes in constraints (see Methods). As expected, the distribution of the likelihood-ratio test statistic followed a chi-squared distribution, and the p-values are uniformly distributed (Figure S2A, grey columns). Next, because short linear motifs are found in rapidly evolving disordered regions that contain indels, we simulated proteins according to the same model but also allowed indels (using INDELIBLE [3], see Methods for indel parameters). We then aligned the proteins (MAFFT, see reference [66] in the main text and Methods), and repeated the likelihood-ratio test for changes in constraints. The inclusion of indels in the simulation led to an increased rejection rate of the null hypothesis (Figure S2A, black circles), presumably because alignment errors lead to analysis of non-homologous residues and the extra rate parameter in the alternative hypothesis can 'fit' some of this heterogeneity. However, after performing the non-central correction to the chi-squared distribution, the distribution of p-values was uniform, indicating that the indel and alignment process was adequately captured by the non-central parameter (Figure S2A, white circles). The KL divergence for this particular set of parameters was 0.000837.

To test whether the non-central correction could account for heterogeneity in the substitution process, we next performed codon-based simulations where we could vary the stationary codon

frequencies. We simulated proteins according to a codon model (with Ka/Ks is equal to 1) using the codon frequency table from *Thermus aquaticus* (which is GC-biased), and found that the likelihood-ratio test statistic followed a chi-squared distribution (Figure S2B, grey columns) despite the GC-biased codon model likely leading to amino acid frequencies different than those assumed by the WAG model in the test. Next, we simulated a similar set of proteins, except that one of the tested clades was evolving under the *S. cerevisiae* codon frequency table (which is AT-biased). Because the substitution model changes on the phylogeny, this set of proteins corresponds to proteins evolving under a non-homogenous and non-stationary process. As expected, we observed increased false rejections of the null hypothesis (Figure S2B, black circles) because the alternative hypothesis can account for some of this heterogeneity using the additional rate parameter. However, after correction by the non-central parameter, the p-values were now uniformly distributed (Figure S2B, white circles). The KL divergence for this set of parameters was 0.001031.

To illustrate how all these deviations of the evolutionary models can be compounded in the analyses, we also evolved proteins under the same non-homogenous, non-stationary processes, but now also included indels (see Methods). Doing this, we observed a much higher false rejection rate of the null hypothesis (Figure S1B, black squares); however, even while several factors compounded to deviate from the models assumed in the test, it was still possible to capture the deviation to the null hypothesis using a single non-central parameter (Figure S1B, white squares). Only a very small increase in the KL divergence for the combined deviations to the model was observed (0.001059 vs 0.001031 for the heterogeneous substitution model with no indels); however, we observed a much higher false rejection rate of the null-hypothesis because

the likelihood-ratio test is performed on more columns (more data points in the test) due to the indels.

Taken together, these results indicate that a non-central chi-squared distribution can be used to capture some of the evolutionary complexities that can be encountered when detecting functional divergence after gene duplication.

## Supplementary Methods

### Simulation of protein evolution under various evolutionary models

To simulate short linear motif evolution of different lengths (Figure S1), we estimated a phylogenetic tree from Cdc20 (a protein conserved in all eukaryotes) using AAML and under the global clock model. We used this tree with the program INDELIBLE [3] to evolve short sequences of different lengths under the WAG model. These sets of simulated short linear motifs therefore correspond to the null model of the likelihood-ratio test.

To simulate protein sequences under various models of evolution for the purpose of testing the non-central chi-squared correction to the likelihood-ratio test (Figure S1), we used the program INDELIBLE [3]. The same phylogenetic relationship was always used: (((a,b),(c,d)),((e,f),(gh))) with equal branch lengths of 0.8 and a root length of 300 (codons or amino acids). We arbitrarily set the (e,f) clade to be the post-WGD clade and performed likelihood-ratio tests on proteins simulated by the program. Sequences were evolved with the following parameters:

1) WAG model,

2) WAG model with power law distributed indels, with a=1.7 for inserts, and a=1.8 for deletions, with an indel rate of 0.1,

3) Homogeneous codon models with codon stationary frequency equals to the *Thermus aquaticus* codon frequency with kappa=2, omega=1,

4) Non-homogeneous codon models, and codon stationary frequency equals to the *S. cerevisiae* codon frequency for all the tree, except for the (e,f) clade which was set to the *Thermus aquaticus* codon frequency,

5) same as 4) except with indels similar to test 2) except with indel rate of 0.05.

The likelihood-ratio test was then performed on the resulting proteins (or aligned first with MAFFT if indels were simulated).

**Strains and plasmids**

BY4741 or isogenic derivatives were used for all of our experiments. Single-copy genes were PCR amplified from purified genomic DNA (Fermentas, #K0512) of *L. kluyveri* (NRRL Y-12651) and *L. waltii* (UCD 72-13), and Ace2/Swi5 orthologs were PCR amplified from purified DNA from *C. glabrata* (CBS 138). Allele replacement for single-copy genes was performed using a modification of the method as in [4] with single-copy genes replacing the *SWI5* gene. Briefly, the 3'UTR of *ACE2* or *SWI5* was cloned into pFA6a [5] (PCR primers P7/P8 and P9/P10 respectively), after which genes of interests were cloned upstream (Ace2(L.klu) – PCR primers P34/P35; and Ace2(L.wal) – PCR primers P36/P37). Two PCR fragments were then transformed to target the *SWI5* locus (PCR primers CaURA3MX: P29/P44, Δ*swi5*::*ACE2(L.klu)* P32/P31, Δ*swi5*::*ACE2(L.wal)* P46/P31). The selection marker used for our experiments was the

CaURA3MX cassette, which allowed subsequent marker removal using 5-FOA [6]. Once the marker was removed, all strains contained precise gene replacement of *SWI5* and these were then tagged with monomeric yeast-enhanced GFP using the same method as in reference [45] from the main text with either the CaURA3MX or KanMX4 resistance marker instead of the HIS3MX4 (Ace2(S.cer) [YBS31] – PCR primers P5/P41, Swi5(S.cer) [YBS32] – PCR primers P2/P44, Ace2(L.klu) [YBS14] – PCR primers P12/P44 and Ace2(L.wal) [YBS13] – PCR primers P11/P44). For the *C. glabrata* orthologous genes, gene tagging and allele replacement was performed in a single step by transforming two fragments: one containing the gene with homology to the S288C genome on the 5' end and to the GFP cassette on the 3' end, and one containing the GFP with homology to the S288C genome on the 3' end (Δ*ace2*::*ACE2(C.gla)* [YBS17] – PCR primers P38/P40 and P40/P41, and Δ*swi5*::*SWI5(C.gla)* [YBS18] – PCR primers P42/P43 and P40/P41). All strains were verified using genomic PCR and sequencing of the homologous recombination junctions.

All primer sequences and strains used for these experiments are included in Table S2.

Strains were then imaged by growing the cells to log-phase in minimal defined media with appropriate auxotrophic requirements and imaged with a standard 491nm blue laser on a Leica spinning-disc confocal microscope.

## Supplementary references

1. Zhang J, Nielsen R, Yang Z (2005) Evaluation of an improved branch-site likelihood method for detecting positive selection at the molecular level. Mol Biol Evol 22: 2472–2479. doi:10.1093/molbev/msi237.

2. Yang Z, dos Reis M (2011) Statistical properties of the branch-site test of positive selection. Mol Biol Evol 28: 1217–1228. doi:10.1093/molbev/msq303.

3. Fletcher W, Yang Z (2009) INDELible: a flexible simulator of biological sequence evolution. Mol Biol Evol 26: 1879–1888. doi:10.1093/molbev/msp098.

4. Li Z, Vizeacoumar FJ, Bahr S, Li J, Warringer J, et al. (2011) Systematic exploration of essential yeast gene function with temperature-sensitive mutants. Nat Biotechnol 29: 361–367. doi:10.1038/nbt.1832.

5. Wach A, Brachat A, Pöhlmann R, Philippsen P (1994) New heterologous modules for classical or PCR-based gene disruptions in Saccharomyces cerevisiae. Yeast Chichester Engl 10: 1793–1808.

6. Boeke JD, Trueheart J, Natsoulis G, Fink GR (1987) 5-Fluoroorotic acid as a selective agent in yeast molecular genetics. Methods Enzymol 154: 164–175.
