# Supplementary figures and images for: Detecting Functional Divergence after Gene Duplication through Evolutionary Changes in Posttranslational Regulatory Sequences
Source: PLoS Comput Biol. 2014 Dec 4;10(12):e1003977. doi: 10.1371/journal.pcbi.1003977 (PMC4256066; doi:10.1371/journal.pcbi.1003977)

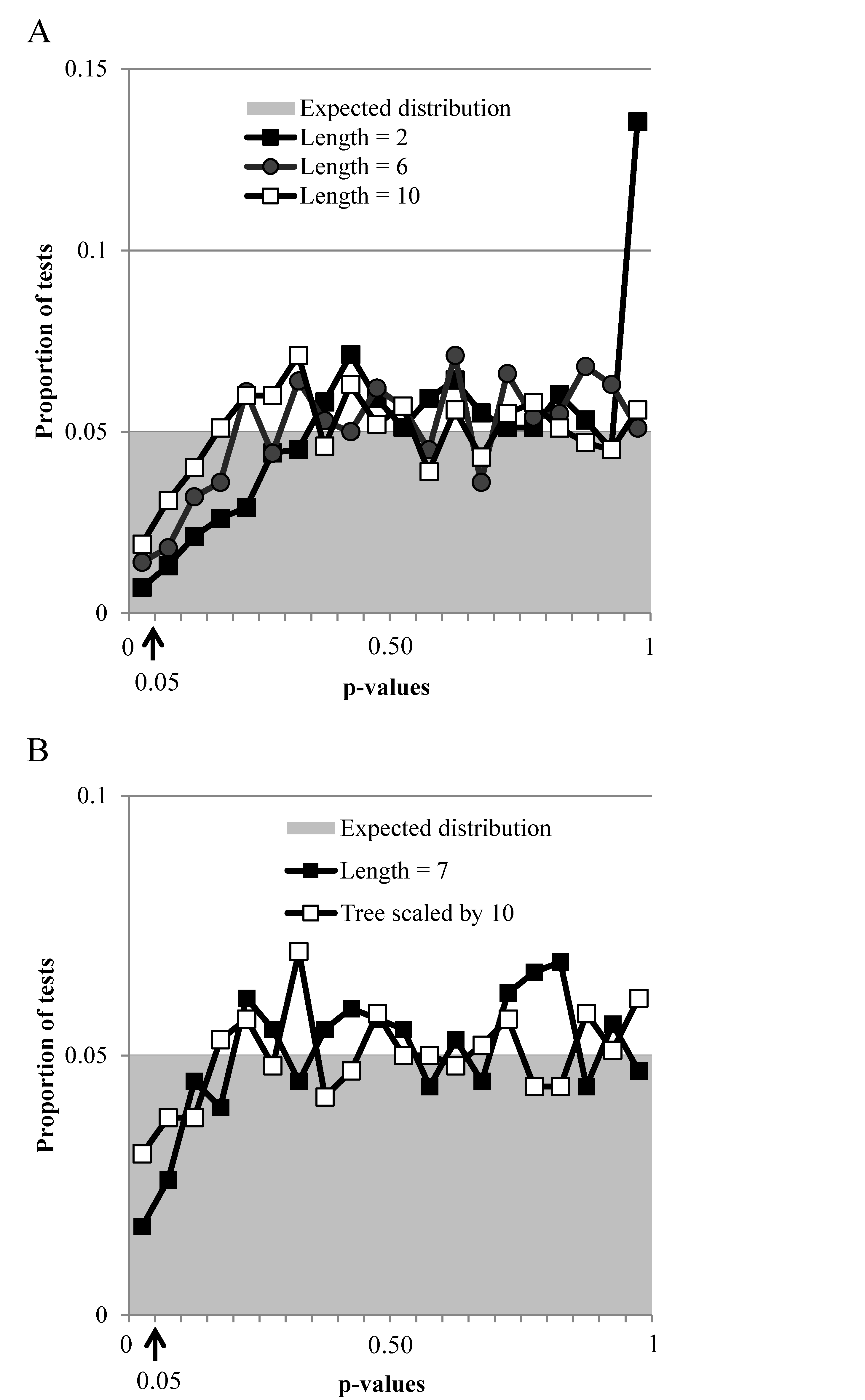

Supplement: Figure S1 — The chi-squared approximation of the distribution of likelihood-ratio test on short sequences is conservative. A) Short amino acid sequences of various lengths were evolved under the WAG model with the same phylogenetic tree that follows a global clock (corresponding to the null model assumed by the test). Grey bars show the expected distribution of p-values if the chi-squared approximation is correct. Data points are the obtained distribution of p-values. B) Short linear motifs of length 7 were evolved using the same procedure as in A) but the phylogenetic tree was scaled to allow for more substitutions per sites, showing that more substitutions do not lead to more false rejections than expected for short sequences. (TIF) [file pcbi.1003977.s002.tif]

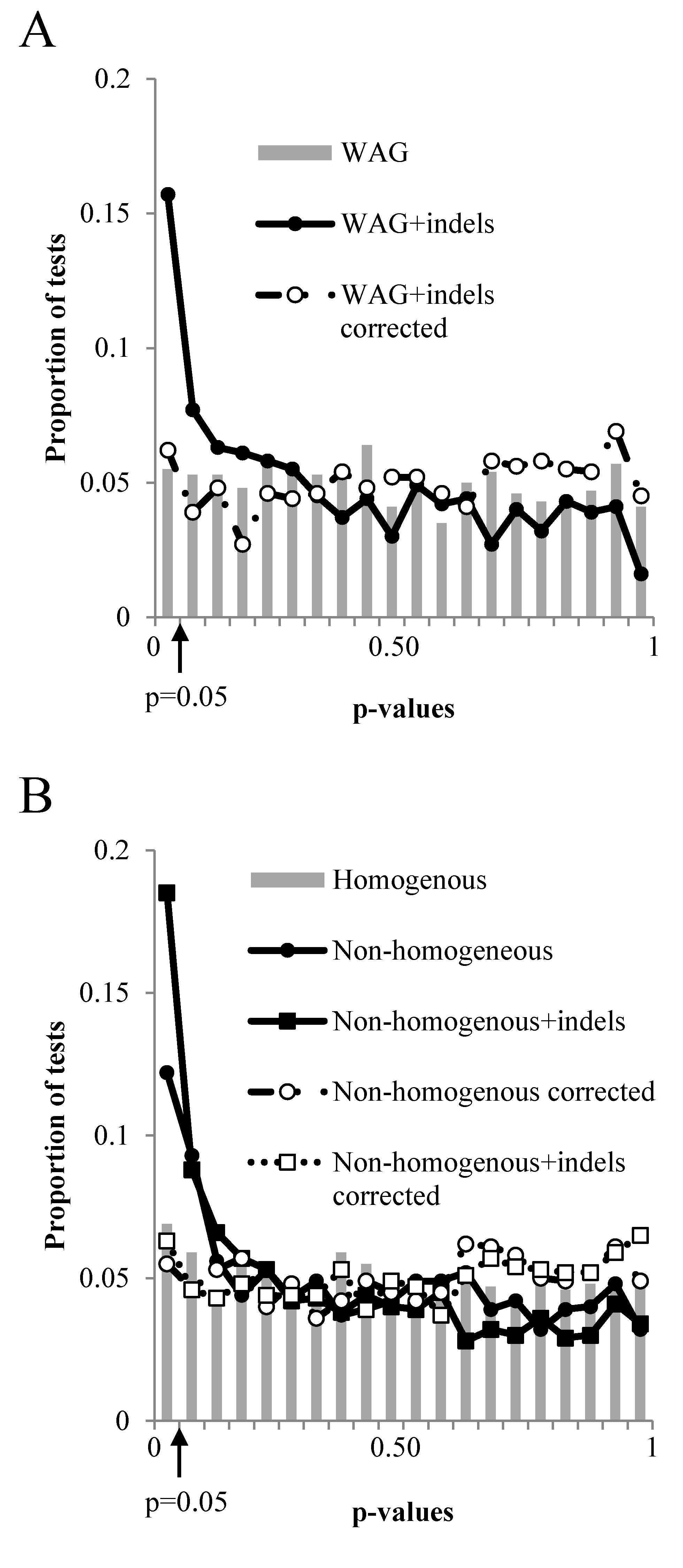

Supplement: Figure S2 — P-value distribution of the likelihood-ratio test obtained from chi-squared and non-central chi-squared on simulated data. A) Amino acid sequences were evolved under the WAG model with or without indels. Grey bars show the distribution of p-values obtained from the likelihood-ratio test when the data are generated according to the model assumed by the test. Circles indicate the distribution of p-values when indels are also included and data is aligned, and the test statistic is assumed to be chi-squared distributed (black circles) or non-central chi-squared distributed (white circles, “corrected”). B) Protein coding DNA sequences were evolved. Grey bars show the distribution of p-values when sequences are evolved under a homogenous and stationary codon frequency model assumed by the test. Circles indicate the distribution of p-values when the model is non-homogenous and the test statistic is assumed to be chi-squared distributed (black circles) or non-central chi-squared distributed (white circles, “corrected”). Squares indicate the distribution of p-values when the indels are also included and the test statistic is assumed to be chi-squared distributed (black squares) or non-central chi-squared distributed (white squares, “corrected”). (TIF) [file pcbi.1003977.s003.tif]

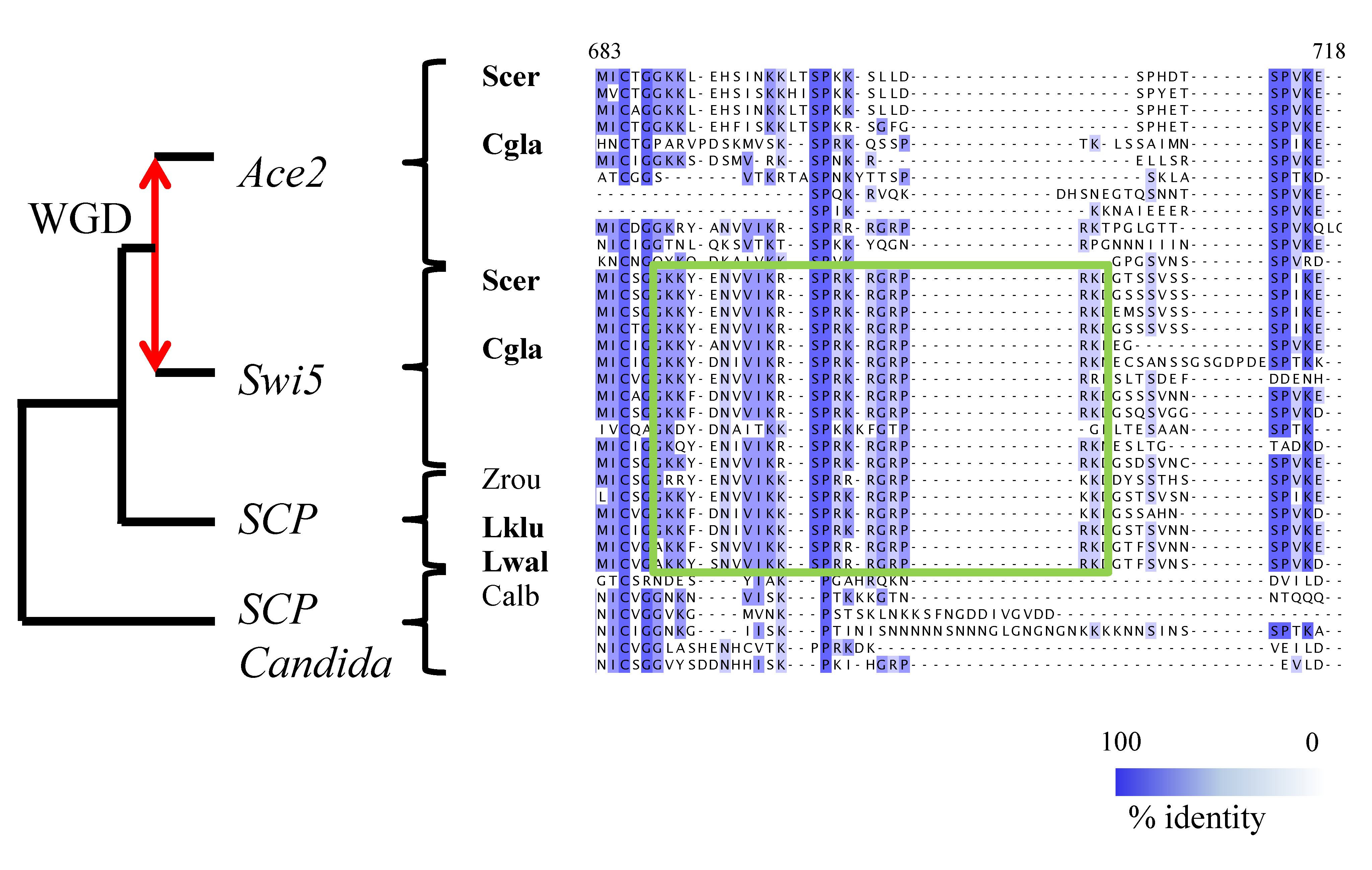

Supplement: Figure S3 — Alignment of the characterized nuclear localization signal of Swi5 and other species. Green box indicates the characterized nuclear localization signal in the Swi5 lineage. Coordinates of Ace2 are indicated in the alignment. Bolded species indicate species that we tested for their localization in Fig. 5. (TIF) [file pcbi.1003977.s004.tif]
